# Reconstructing mitochondrial genomes directly from genomic next-generation sequencing reads—a baiting and iterative mapping approach

**DOI:** 10.1093/nar/gkt371

**Published:** 2013-05-09

**Authors:** Christoph Hahn, Lutz Bachmann, Bastien Chevreux

**Affiliations:** ^1^Natural History Museum, University of Oslo, Oslo N-0318, Norway and ^2^Department for Human Nutrition and Health, DSM Nutritional Products Ltd., Kaiseraugst CH-4303, Switzerland

## Abstract

We present an *in silico* approach for the reconstruction of complete mitochondrial genomes of non-model organisms directly from next-generation sequencing (NGS) data—mitochondrial baiting and iterative mapping (MITObim). The method is straightforward even if only (i) distantly related mitochondrial genomes or (ii) mitochondrial barcode sequences are available as starting-reference sequences or seeds, respectively. We demonstrate the efficiency of the approach in case studies using real NGS data sets of the two monogenean ectoparasites species *Gyrodactylus thymalli* and *Gyrodactylus derjavinoides* including their respective teleost hosts European grayling (*Thymallus thymallus*) and Rainbow trout (*Oncorhynchus mykiss*). MITObim appeared superior to existing tools in terms of accuracy, runtime and memory requirements and fully automatically recovered mitochondrial genomes exceeding 99.5% accuracy from total genomic DNA derived NGS data sets in <24 h using a standard desktop computer. The approach overcomes the limitations of traditional strategies for obtaining mitochondrial genomes for species with little or no mitochondrial sequence information at hand and represents a fast and highly efficient *in silico* alternative to laborious conventional strategies relying on initial long-range PCR. We furthermore demonstrate the applicability of MITObim for metagenomic/pooled data sets using simulated data. MITObim is an easy to use tool even for biologists with modest bioinformatics experience. The software is made available as open source pipeline under the MIT license at https://github.com/chrishah/MITObim.

## INTRODUCTION

Mitochondrial DNA is a useful and particularly popular marker in molecular ecology, population genetics, evolutionary biology, as well as in phylogeographic and phylogenetic studies of animals (1–4). High mutation rate, lack of recombination, maternal inheritance, high copy number and therefore relatively easy accessibility often makes mitochondrial DNA the molecular marker of choice. In the early years, only relatively short mitochondrial regions were targeted, but with improving methodology, sequencing of complete mitochondrial genomes became more common, even when exploring difficult templates such as ancient DNA. Complete mitochondrial genomes are particularly useful when attempting to answer long-standing questions of human evolution (5,6) or to explore the evolutionary histories of enigmatic species such as the Mammoth, Aurochs, Tasmanian tiger or Polar bear (7–11).

Until recently, sequencing of mitochondrial genomes was a somewhat challenging and resource demanding task. It has been approached using the conventional strategy of combining long-range PCR with subsequent primer walking [e.g. (12–18)]. The paradigm shift caused by recently developed next-generation sequencing (NGS) technologies lead to the proposal of more straightforward integrated pipelines for sequencing complete mitochondrial genomes (19). The long-range PCR strategy nevertheless remained an integral method for the majority of recent studies [e.g. (20,21)], especially to overcome many NGS platforms demand for moderate-to-high amounts of template DNA, which is of particular concern when dealing with limited material such as small invertebrates (22). However, optimizing long-range PCR can be tedious, in particular for previously uncharacterized organisms when the information required for primer design is scarce, whereas for difficult templates such as ancient DNA, long range PCR is no option at all, given the level of DNA degradation. Approaches using exclusively NGS without prior long-range PCR have not yet become standard procedure because the bioinformatics for reliable sequence assembly of mitochondrial genomes from genomic data is not trivial.

For well-known model organisms, with mitochondrial reference genomes available, capture protocols and subsequent NGS sequencing of enriched DNA templates is a highly efficient, yet challenging, approach (23,24). Mitochondrial genomes of previously sequenced, but less well-established, organisms can be targeted by mapping assembly approaches, where the available reference is used as a backbone, guiding the finding and assembling of relevant reads from the genomic readpool for new geographic variants/strains of the organism in question. In the absence of closely related reference sequences, this strategy is however problematic. Mapping approaches require a meaningful stringency setting to limit the number of tolerable mismatches in the sequence comparisons. This is first of all to avoid inclusion of false positive reads that will cause conflicts in the downstream assembly process. A reference sequence from a too distantly related species will thus only allow to identifying reads mapping to the highly conserved regions in the target species. *De novo* assembly, i.e. the reconstruction of genomic sequences without any reference information has, due to the exponential increase of accessibility to NGS data, become an increasingly important and dynamic field of research. Sequence assembly algorithms are continuously being developed [e.g. (25–29)], improved and assessed (30,31). It is, however, impossible at the NGS read level to distinguish between nuclear and mitochondrial sequences, and the similarity of genuine mitochondrial sequences and nuclear copies of mitochondrial (NUMT) DNA may cause conflicts for the assembly software. Furthermore, the high copy number of mitochondrial DNA and the resulting high coverage will worsen the signal-to-noise ratio, rendering a read assembly far from straightforward.

Here, we present a straightforward approach for reconstructing novel mitochondrial genomes directly from NGS data generated from total genomic DNA extracts. We use real data obtained from monogenean ectoparasitic flatworms of the genus *Gyrodactylus* (and their teleost fish hosts) as case studies for the proposed mitochondrial baiting and iterative mapping (MITObim) approach. We demonstrate how MITObim is capable of assembling mitochondrial genomes without the need of a reference genome of the targeted species by relying solely on mitochondrial genome information of more distantly related taxa as a starting point, and compare its performance to the previously published tools Mapping Iterative Assembler (MIA) (5) and Iterative Mapping and Assembly for Gap Elimination (IMAGE) (32). Most importantly, we demonstrate the novel approach of reconstructing complete mitochondrial genomes using exclusively cytochrome-oxidase subunit 1 (COI) sequences, i.e. the commonly used mitochondrial DNA barcode for animals (33), as seed references. The high specificity of MITObim, emphasized in a case study using simulated data, makes it applicable for metagenomics and facilitates the sequencing and subsequent specific assembly of mitochondrial genomes from mixed samples containing different species.

## MATERIALS AND METHODS

### Molecular methods

A total of 100 *Gyrodactylus thymalli* and 100 *Gyrodactylus derjavinoides* individuals collected from hatchery reared host fish European grayling (*Thymallus thymallus*) and Rainbow trout (*Oncorhynchus mykiss*), respectively, were separately pooled for DNA extraction using the E.Z.N.A. Tissue DNA kit (Omega Bio-Tek) following the Tissue DNA - Spin Protocol. Genomic DNA was prepared in paired-end libraries, tagged and subjected to NGS on the illumina HiSeq 2000™ (outsourced to GENterprise GENOMICS, Mainz, Germany).

### Pre-processing of NGS data

NGS reads (read length 100 bp) were error corrected using the error correction tool (personal communication Ruibang Luo, BGI) of the SOAPdenovo2 software (34) and quality trimmed using Mimicking Intelligent Read Assembly (MIRA) v3.4.1.1 [(35), http://sourceforge.net/projects/mira-assembler/] for subsequent analyses. Details can be found in Table 1.

**Table 1.** Read statistics pre and post error correction and trimming for the two samples sequenced using an illumina HiSeq 2000™ instrument

Sample name	Parasite	Host	Amount genomic DNA (µg)	No. of bases (Gb)	Insert size	Post error correction and trimming	
						No. bases (Gb)	Average readlength
thy	*G. thymalli*	*T. thymallus*	3.3	4.25	242 ± 73	2.87	97
der	*G. derjavinoides*	*O. mykiss*	2.4	3.46	234 ± 85	1.72	95

The comparatively high losses for illumina type sequencing data are due to the rigorous removal of reads representing low coverage genomic data by error correction and trimming routines.

### Algorithm overview

**Figure 1.**
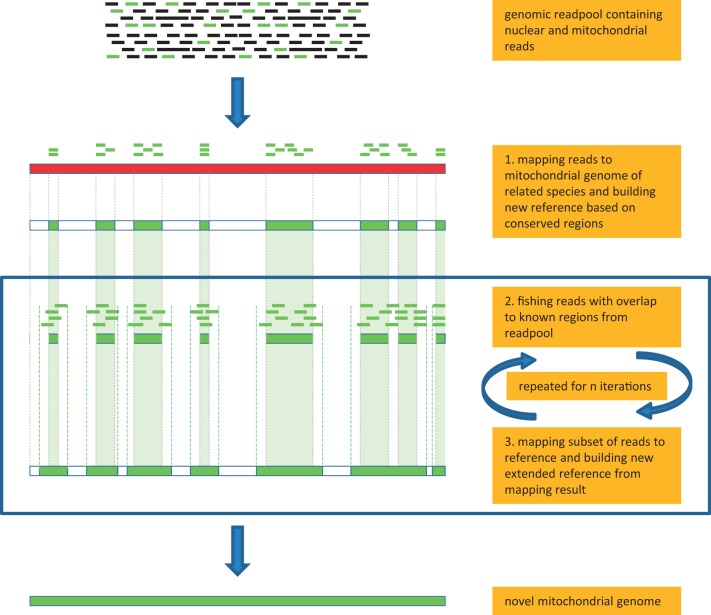
Schematic workflow of MITObim procedure. Step one, mitochondrial reads are mapped to conserved regions on related reference sequence. An initial reference for the species in question is build from the mapping result. Step two, fishing reads with overlaps to previously identified regions from the readpool. Step three, mapping this subset of reads and creating new extended reference. Steps two and three are iteratively repeated until all gaps are closed and the number of reads remains stationary. Black rectangles, nuclear reads; red rectangle, mitochondrial genome of distantly related species; green rectangles, mitochondrial reads and growing mitochondrial reference.

The workflow of the MITObim approach is schematically illustrated in Figure 1. All steps are performed using modules of the MIRA sequence assembler software (35): the main assembly module (MIRA v3.4.1.1), which is used in mapping mode to map reads to a reference and create new reference sequences; and an *in silico*-baiting module (MIRAbait, see http://mira-assembler.sourceforge.net/docs/DefinitiveGuideToMIRA.html#sect_mutils_bait for more information), which is used to extract reads that precisely match a given reference across a number of n k-mers of length k (defaults *n* = 1 and k = 31) from the entire set of reads. In brief, step one identifies conserved regions between genomic reads of the target organism to the mitochondrial genome of a reference species by a mapping assembly from which a new reference is derived. This new reference may initially be gapped or consist of several *contigs*, as non-conserved parts of the target organism will not have mapped to the mitochondrial genome of the reference species. Alternatively, step one can facilitate a mitochondrial barcode sequence of the target species or a close relative as initial reference. Step two of MITObim is an *in silico*-baiting step: using the newly created reference sequence as bait, all reads from the total genomic readpool, which are partly or fully overlapping with the bait (minimum overlap is n k-mers of length k) are retrieved and integrated into a new data set. This baiting step with relatively long k-mers (k = 31) is crucial for the specificity of the whole process as the subsequent mapping assembly is performed with comparatively low stringency, i.e. allowing mismatches in up to 15% of the bases of the total length of a Smith–Waterman alignment overlap. Furthermore, the substantial reduction of the readpool decreases downstream CPU time and memory requirements. In the third step, the reads identified in the previous step are mapped back to the gapped reference sequence using MIRA v3.4.1.1. This will lead to an extension of the reference sequence and a reduction of gaps. To be incorporated as an extension into a mapping assembly, a read requires an overlap of at least 30 bases at the edge of a reference. Read pair information in mapping assemblies can therefore not increase the rate of *contig* extension per iteration but can be used to increase assembly accuracy as implemented in the proofreading algorithm of MITObim (see Supplementary Methods). If data sets can be expected to be ‘well behaved’, however, i.e. in the absence of contamination by closely related species and for mitochondrial genomes without repeat regions, a less conservative approach can be chosen as an initial strategy: MITObim allows to replace the mapping assembly by a *de novo* assembly, which may optionally facilitate read pairing information, i.e. incorporate the mates of all baited reads into the readpool. All these reads are then assembled *de novo* instead of mapped to the reference. This strategy may decrease the number of necessary iterations (and CPU time) to finish a mitochondrial genome substantially, as it allows to increase the degree of *contig* extension per iteration into the range of the library insert size. Steps two and three are iteratively repeated until the number of mapped reads becomes stationary, i.e. converges. The final iteration results in the complete mitochondrial genome of the target organism build from the iteratively identified mitochondrial readpool either as one or several *contigs*. An optional *de novo* assembly using this reduced readpool may subsequently be performed to confirm the results obtained by MITObim and/or identify potentially problematic regions.

The initial mapping assembly in step one may have increased memory requirements, as it is dealing with the entire genomic readpool. This increased memory consumption can be bypassed by an initial fishing step (-quick option) reducing the readpool to only reads with a certain k-mer overlap to the reference already before the initial assembly. This approach initially slightly decreases specificity, but nevertheless performs well with not too distantly related references available and can be performed as a standard primary test. Where adequate computational resources are available, however, we recommend mapping the entire readpool in step one. All subsequent MITObim steps can be performed on any standard laptop or desktop computer with at least 2 GB RAM, as they deal with a drastically reduced number of reads.

For situations in which only mixed data sets, including mitochondrial genomes from more than one species may be available, e.g. if sample contamination is unavoidable or the data have been generated from a pool of several species to reduce cost for library preparation, a proofreading procedure was developed and implemented in MITObim (at the moment only applicable if a single mitochondrial barcode is to be used as seed reference). Prerequisite for this function is a paired end sequence library. Details about the proofreading procedure can be found in supplementary methods. The MITObim wrapper script is freely available on GitHub (https://github.com/chrishah/MITObim).

### Case study 1—parasitic helminths

In the first case study, we aimed to reconstruct the mitochondrial genomes of the two monogenean ectoparasite species *G. thymalli* and *G. derjavinoides* directly from genomic NGS illumina data using MITObim. The mitochondrial genomes of the two species were downloaded from GenBank as reference sequences (NC_009682 and NC_010976). The data sets ‘thy’ and ‘der’ (see Table 1) were subsequently subjected to MITObim with the *G. derjavinoides* and *G. thymalli* reference sequences as starting point, respectively. Furthermore, the programs MIA (5), developed for obtaining Neanderthal mitochondrial genomes using a *Homo sapiens* reference, and IMAGE (32), developed for gap closing in draft assemblies, were tested on the ‘thy’ data set. MIA v1.0 was run using the *G. derjavinoides* mitochondrial genome (distantly related reference flag was used), whereas IMAGE v2.0 was run on the gapped reference obtained by the initial mapping assembly of the ‘thy’ data set to the *G. derjavinoides* mitochondrial genome using MITObim (–quick option).

### Case study 2—teleost hosts

When sampling *Gyrodactylus* ectoparasites from fish skin, contamination with host tissue is inevitable. Accordingly, the extracted total DNA will not only include genomic DNA from the parasites but also to some extent from the host, i.e. it will resemble a metagenomic sample. However, there is no estimate available about the degree of contamination of such DNA extracts; it can only be assumed that sampling of parasites is done with caution to keep contamination at a lowest possible level. In the second case study, we aimed at using the contaminating host sequences to demonstrate MITObim for the mitochondrial genomes of the teleost fish hosts European grayling (*T. thymallus*) and Rainbow trout (*O. mykiss*), i.e. the respective hosts of the parasite samples leading to the data sets ‘thy’ and ‘der’. The mitochondrial genomes of four salmonids Atlantic salmon (*Salmo salar*), Arctic charr (*Salvelinus alpinus*), European grayling (*T. thymallus*) and Rainbow trout (*O. mykiss*) were downloaded from GenBank (AF133701, NC_000861, FJ853655 and DQ288270). The data set ‘thy’ was subjected to MITObim using the mitochondrial genomes of *S. salar*, *S. alpinus* and *O. mykiss*, whereas for the data set ‘der’, the mitochondrial genomes of *S. salar*, *S. alpinus* and *T. thymallus* were used as starting references.

### Case study 3—COI barcode seeds

The mitochondrial COI gene is a popular barcoding marker for molecular taxonomy (33) of animals, and therefore COI sequences are available for a vast number of species. We assembled the mitochondrial genome of *T. thymallus* directly from the genomic data set ‘thy’ using only a ∼650 bp *T. thymallus* COI sequence downloaded from GenBank (HQ961017) as a seed reference for a subsequent MITObim assembly. For this application, MITObim was run both in mapping and *de novo* assembly mode (see ‘Algorithm Overview’ section).

### Case study 4—simulated metagenomic data

Paired-end illumina data sets (read length 150 bp, insert size 300 ± 50 bp, error rate 0) were simulated for five salmonid mitochondrial genomes, i.e. the four genomes used in case study 2 and the mitochondrial genome of *Salmo trutta* retrieved from GenBank (NC_010007), to a coverage of ∼50× (i.e. 6000 reads per reference) using wgsim from SAMTOOLS (36). A single pooled data set containing all generated reads was created from the simulated data. Five individual MITObim instances were subsequently run (in proofreading mode), each using a ∼1200 bp COI barcode as initial seed for the reconstruction of individual mitochondrial genomes from the pooled data set. Seeds were obtained from the mitochondrial genomes of the targeted species, respectively, as the proofreading procedure requires 100% sequence identity for successful incorporation of reads into the growing readpool (see Supplementary Methods).

### Quality assessment of the MITObim performance

To evaluate the MITObim results, we performed direct mapping assemblies to the correct reference genomes using MIRA v3.4.1.1 (35). All mapping results were visualized and quality checked using the program Tablet (37). *De novo* assemblies on the mitochondrial readpools identified by MITObim were performed by MIRA v3.4.1.1 (35), Velvet v1.2.07 (25) and ABySS v1.3.3 (26) to further verify the results obtained by MITObim. All obtained consensus sequences were aligned to the respective reference using Clustal W (38). Any 5′ and/or 3′ overhangs in respect to the reference were removed and/or displaced in MEGA 5 (39) after verification of correct circularity. Resulting sequences were subjected to Blast searches (40) against GenBank to confirm identity and subsequently compared with each other using Blast (41). The MITOS web server (42) was used for automated annotation of the obtained mitochondrial genomes.

Scripts from the software package khmer (43) were used to calculate k-mer frequencies (20 mer) for (i) the entire data sets ‘thy’ and ‘der’ and (ii) for the parasite mitochondrial readpools identified by MITObim, to estimate the mitochondrial copy number relative to the number of nuclear copies based on the relative k-mer frequency distributions. The parasites’ nuclear genome size was estimated based on the k-mer frequency distributions of the entire data sets, respectively, as demonstrated in previous studies (44,45).

## RESULTS

### Case study 1—parasitic helminths

Supplementary Figure S1 illustrates the progress of the MITObim procedure for the example of the mitochondrial genome of *G. thymalli* from the ‘thy’ readpool using the mitochondrial genome of *G. derjavinoides* as starting reference. The initial mapping assemblies starting the MITObim process identified 56.7% of the *G. derjavinoides* and 52.3% of the *G. thymalli* mitochondrial genomes as conserved enough for a meaningful mapping of reads from the ‘thy’ and ‘der’ readpool. Subsequently, MITObim required 15 and 12 iterations to complete the mitochondrial genomes of *G. thymalli* and *G. derjavinoides* into a single *contig*, respectively. A total of 322 037 ± 483 and 59 976 ± 626 reads were identified as being of mitochondrial origin by direct mapping and MITObim reconstruction. This represents 0.76 and 0.17% of the total genomic readpool and results in an average per base coverage of 2112× and 387× of the mitochondrial genomes of *G. thymalli* and *G. derjavinoides*, respectively. *De novo* assemblies using the identified mitochondrial readpools resulted in only fragmented representations of the mitochondrial genomes of both species. In comparison, using the entire ‘thy’ data set, MIA ran into memory issues (crashed on a 40 Gb RAM machine). To allow for a minimum comparison with MITObim, we simplified the task for MIA by repeating the run using exclusively the mitochondrial reads of *G. thymalli* previously identified by MITObim.

To estimate genome sizes and approximate mitochondrial copy numbers, we used k-mer distributions. For the ‘thy’ data set, the distributions peaked at values of 13 for the entire data set and at 1687 for the mitochondrial readpool identified by MITObim. This indicates a ∼130-fold excess of mitochondrial copies compared with nuclear copies in the ‘thy’ data set. A k-mer coverage distribution based estimation for the genome size of *G. thymalli* yielded ∼170 Mb. Applying the same method exclusively to the mitochondrial reads results in an estimated mitochondrial genome size of ∼14 890 bp. In case of the data set ‘der’, the coverage peak representing the nuclear genome could not reliably be distinguished from unspecific noise occurring at low k-mer coverage levels (most likely caused by random sequencing errors) and was therefore not used for further analyses.

### Case study 2—teleost hosts

On average, 81 and 85% of the hosts’ mitochondrial genomes were identified in the initial mapping steps in the course of the MITObim procedure for *T. thymallus* and *O. mykiss* (i.e. data sets ‘thy’ and ‘der’), using the mitochondrial genomes of the three heterologous species as initial reference, respectively. In the case of *S. alpinus* as reference for ‘thy’, a highly increased read coverage was detected in a ∼100 bp region. Close examination revealed a case of sequence similarity between *S. alpinus* and *G. thymalli*, causing the coverage in this region to increase to ∼1000× compared with ∼50× across the remaining reference. The initial mapping assembly was thus repeated with higher stringency to avoid false positives, reducing the initially identified portion of the genome to a conservative 38%. Accordingly, the subsequent MITObim processes, using the three different mitochondrial references, required on average 8 and 5 iterations to complete the mitochondrial genomes of *T. thymallus* and *O. mykiss*. Direct mapping approach and MITObim identified 8876 ± 20 and 3151 ± 5 reads in the mitochondrial readpool of the hosts *T. thymallus* and *O. mykiss*, respectively, representing 0.02 and 0.01% of the total genomic readpool and an average coverage of 53× and 18×. Total host contamination in the data sets ‘thy’ and ‘der’ can therefore be estimated to 3 and 5%, respectively.

### Case study 3—COI barcode seeds

In this case study, both the standard mapping and the modified MITObim strategies yielded identical sequences. The standard strategy, however, required a total of 115 iterations to complete, whereas the modified approach using *de novo* assembly including paired end information, reached a stationary read number already after 26 iterations. The number of mitochondrial reads identified for *T. thymallus* in this case study was 8885 ± 2, equivalent to a 53× average coverage.

### Case study 4—simulated metagenomic data

Using the proofreading option, MITObim individually recovered all five mitochondrial genomes included in the pooled data set. In all cases, the accuracy exceeded 99% and >96% of the reads simulated for a respective mitochondrial genome were recovered, whereas the false positive rate, i.e. incorporating heterologous reads into the readpool, was on average below 0.5%. The length of the recovered mitochondrial genomes in general exceeded 99.5% of the reference. In case of the *S. salar* data set, MITObim halted a further extension at ∼97% of the reference length owing to conflicts with reads originating from the congener *S. trutta*. Table 3 summarizes the results for this case study.

### Quality assessment of the MITObim results

Blast searches of the mitochondrial genomes obtained by MITObim returned in all cases the mitochondrial genomes of the targeted species as best hit and the MITObim genomes as well as the genomes obtained by direct mapping had a sequence identity exceeding 99.5% (see Table 2). Two kinds of errors were observed: (i) Single nucleotide substitutions and (ii) short gaps, i.e. insertions/deletions (indels).

**Table 2.** Summary of the quality assessment for case studies 1 to 3

Sample name	Case study 1		Case study 2				Case study 3
	*G. thymalli*	*G. derjavinoides*	*T. thymallus*	*O. mykiss*	*S. salar*	*S. alpinus*	*T. thymallus* (COI)
thy		8/4 (99.87%)		0/0 (100%)	0/0 (100%)	0/0 (100%)	0/0 (100%)
der	8/7 (99.62%)		0/1 (99.99%)		0/0 (100%)	0/1 (99.99%)	

Number of single nucleotide substitutions/indels (percentage identity) is given for the used starting reference/seed in respect to the direct mapping result using the correct mitochondrial reference sequence.

**Figure 2.**
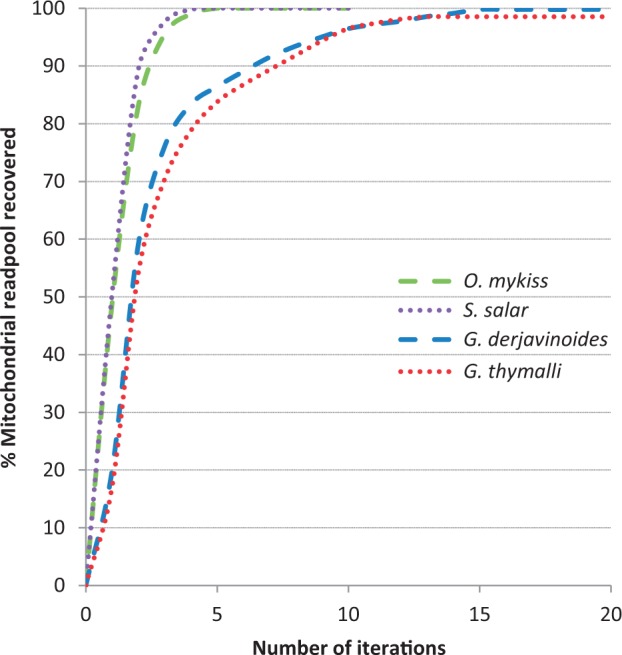
Number of MITObim iterations to reach a stationary mitochondrial read number depending on the initial reference used. Data sets ‘thy’ and ‘der’ are represented by dashed and dotted lines, respectively. Initially used reference is indicated by color.

All runs, except the standard MITObim in case study 3, yielded mitochondrial genomes in <24 h. The number of iterations necessary to close all gaps and to reach a stationary number of reads in the mitochondrial readpool varied depending on which reference sequence was used (see Figure 2). In general, MITObim performed better on the teleost host genomes than on the *Gyrodactylus* genomes (for details, see Table 2). The assembly of the parasite genomes required more iterations to complete. Web-based automated annotation identified all mitochondrial genes and tRNAs as reported for the respective GenBank entries for the mitochondrial genomes obtained using MITObim. No differences in gene order were found between direct mapping and MITObim results. Using the simplified data set of exclusively the mitochondrial readpool, MIA recovered a mitochondrial genome of 99.24% base accuracy containing 95 single nucleotide substitutions (including unclosed gaps) and three indels. Still, the necessary CPU time for MIA exceeded the MITObim runtime by a factor of 15 (1.05 h MITObim versus 15.75 h MIA). For IMAGE, we failed to find a parameter combination, which would have allowed to close any gaps of the initially constructed reference.

## DISCUSSION

Improvements in sequencing technologies have made genomic data increasingly available to researchers, and NGS projects are easily feasible for non-model organisms. Mitochondrial genomes will certainly remain paramount markers for addressing various biological questions, yet obtaining novel mitochondrial genomes is for the most part still labor and resource intensive. The proposed MITObim approach aims at providing an easy to use tool to assemble mitochondrial genomes of non-model organisms directly from NGS data. An initial identification of mitochondrial seed reads can be guided by either mitochondrial genomes of (distantly) related species or commonly available mitochondrial barcoding marker sequences, in animals usually the COI gene, of the species in question.

The genetic distance between the available reference and targeted species is directly affecting the required number of iterations for successfully completing the mitochondrial genome (Figure 2). The average genetic K2P distance between the two *Gyrodactylus* species used in case study 1 is 0.26, whereas the average distance between the four salmonids species used in case study 2 is only 0.13. Genetic distance between *Gyrodactylus* ectoparasites represents an extreme case of genetic divergence within a genus comparable with the distance between Human and Macaque (K2P *H. sapiens* versus *Macaca sylvanus* ≈ 0.25). The portion of the mitochondrial genomes identified in the initial mapping assembly and the number of MITObim iterations necessary to complete a mitochondrial genome from a given starting reference varied accordingly.

The mitochondrial genomes of the studied gyrodactylid flatworms show a peculiar ca 750–800 bp long non-coding repeat region (12,13). The MITObim errors reported in Table 2 for case study 1 occur predominantly in these repeat regions. The absence of such repeats reasonably explains the slightly better MITObim performance on the host mitochondrial genomes targeted in case study 2. Repeat regions are a well-known problem for sequence assembly algorithms (25–30,46), and none of the *de novo* assemblers tested managed to assemble the entire gyrodactylid mitochondrial genome from the mitochondrial readpool into just one continuous *contig*. MITObim, however, could close the gaps over the repeat regions with just slightly impaired performance. The few errors outside the repeat regions are associated with homopolymers occurring in regions of high AT-content. For the gyrodactylid and salmonid mitochondrial genomes used in the presented case studies, with an overall AT-content of some 62 and 55%, respectively, this was however not a major issue. The MITObim performance for AT-rich genomes, such as described for some insects, is however yet to be assessed. For most reliable results, we suggest a combination of MITObim and subsequent *de novo* assembly on the reduced readpool to identify potentially problematic regions, which can then be addressed by traditional PCR.

Compared with the previously published program MIA v1.0 (5), MITObim performs slightly better in terms of accuracy while demonstrating a substantial improvement in terms of runtime and memory usage. The software IMAGE v2.0 (33), originally developed for gap closing in draft genome projects, failed the test performed in our study, presumably because the initially identified conserved regions were an insufficient starting point for a successful performance.

For *Gyrodactylus salaris*, a closely related sibling species of *G. thymalli*, NUMT sequences have been postulated although never unambiguously demonstrated (47). In the present study, we did not encounter any problems related to NUMTs. MITObim performance for species with high number of NUMTs still needs to be assessed. We would however not expect this phenomenon to cause problems for MITObim. Although in conventional PCR-based strategies, primer specificity may lead to a serious bias toward the amplification and subsequent sequencing of NUMTs, authentic mitochondrial sequence reads will by a large margin outnumber NUMTs in the MITObim approach.

Case studies three and four demonstrate that the frequently used mitochondrial barcoding marker COI, which is available for a vast number of genetically otherwise unexplored organisms, can be a sufficient seed to retrieve an entire mitochondrial genome. This represents a novel *in silico* approach highly applicable to most animal mitochondrial genomes except for rare cases of mitochondrial genomes containing repeats. For such cases, the mitochondrial genome of a distantly related species will be the more promising reference sequence for completing a novel mitochondrial genome. Large-scale rearrangements of the gene order between the reference mitochondrial genome and that of the organism in question will predictably cause conflicts. However, when starting MITObim from a relatively short seed, this problem will not occur. Case study 4, in particular, demonstrated the applicability and specificity of MITObim for metagenomic data sets or data obtained from mixed samples. The proofreading algorithm implemented (details in Supplementary Methods) potentially enables MITObim to specifically reconstruct mitochondrial genomes, despite shared conserved sub-sequences of a length close to the insert size of the library used for sequencing. The importance of read length and library insert size in case of mixed samples can, however, not be stressed enough (see Supplementary Methods for more discussion in respect to case study 4). The MITObim proofreading procedure will likely introduce assembly errors if the length of conserved regions is exceeding the read library insert size, or halt a further extension, as observed for *S. salar* in case study 4. The incomplete recovery of the remaining mitochondrial genomes in case study 4 (see Table 3) is an artefact of the read simulation from a linear reference, resulting in decreasing coverage toward the end of the reference. In any case, careful inspection of the mapping result in an appropriate viewer (e.g. Tablet) is paramount for detecting potentially problematic regions.

**Table 3.** Results of case study 4

COI reference	No. of iterations	Accuracy (%)	Length (%)	Reads (%)	fp rate (%)
*O.mykiss*	102	100	99.98	99.30	0.27
*S.alpinus*	90	100	99.72	99.53	0.47
*S.salar*	100	99	96.97	96.82	1.14
*S.trutta*	103	100	99.62	99.43	0.57
*T.thymallus*	94	100	99.84	99.47	0.00

For each seed COI sequence, the table illustrates the number of iterations until read number convergence, per base accuracy in percentage, percentage length of the reference mitochondrial genome recovered, fraction of reads recovered from the respective specific readpool and fraction of false positive reads, i.e. reads of heterospecific origin incorporated from the entire mixed readpool.

Based on k-mer coverage distributions, we estimated a 130-fold excess of mitochondrial- relative to nuclear copies for *G. thymalli.* This seems a reasonable estimate; yet, it has to be taken into account that the analysis is based on total genomic DNA extracted from a pooled sample of parasites. The number of mitochondria per cell has been shown to vary substantially across various live stages (48) and tissue types (49). The estimated ∼170 Mb genome size for the monogenean flatworm *G. thymalli* lies within the range reported earlier for other platyhelminthes [e.g. (50,51)]. The estimate of the mitochondrial genome size based on k-mer coverage distribution lies within 1% of the reported mitochondrial genome size for *G. thymalli*, i.e. 14 788 bp (13).

We used real NGS data to demonstrate how the MITObim approach can reconstruct mitochondrial genomes of varying complexity solely based on the mitochondrial genome of more distantly related species as a starting point. MITObim appears superior in runtime, memory consumption and accuracy, when compared with existing tools for this particular application. We furthermore showed the novel application of assembling mitochondrial genomes, particularly in the absence of extensive repeat regions, directly from a short seed sequence, such as the commonly used COI barcode. Finally, we demonstrated the performance of our method to reconstruct mitochondrial genomes from metagenomics data sets in a controlled environment of simulated data. We therefore recognize MITObim as a highly accurate and efficient approach to assembling novel mitochondrial genomes of non-model organisms directly from total genomic DNA derived NGS reads. Labor-intensive long-range PCR steps are no longer required. In principle, MITObim applications can be easily extended to other organelle genomes or even nuclear DNA templates. Its specificity makes it applicable for mixed species samples, and the easy to use wrapper script available makes MITObim a useful tool for biologists with modest bioinformatics experience.

## SUPPLEMENTARY DATA

Supplementary Data are available at NAR Online: Supplementary Figure 1 and Supplementary Methods.

## FUNDING

Internal funding of the Natural History Museum, University of Oslo, Norway. Funding for open access charge: Natural History Museum, University of Oslo, Norway; and Department for Human Nutrition and Health, DSM Nutritional Products Ltd., Switzerland.

*Conflict of interest statement.* None declared.

## Supplementary Material

Supplementary Data
