# Supplementary material for: Reconstructing mitochondrial genomes directly from genomic next-generation sequencing reads—a baiting and iterative mapping approach
Source: Nucleic Acids Res. 2013 May 9;41(13):e129. doi: 10.1093/nar/gkt371 (PMC3711436; doi:10.1093/nar/gkt371)
Supplement: Supplementary Data [file supp_41_13_e129__index.html]

Reconstructing mitochondrial genomes directly from genomic next-generation sequencing reads—a baiting and iterative mapping approach — Reconstructing mitochondrial genomes directly from genomic next-generation sequencing reads—a baiting and iterative mapping approach — Supplementary Data 

# Reconstructing mitochondrial genomes directly from genomic next-generation sequencing reads—a baiting and iterative mapping approach

## Supplementary Data

files

**Files in this Data Supplement:**

- Supplementary Data - pdf file
