# Supplementary material for: Reconstructing mitochondrial genomes directly from genomic next-generation sequencing reads—a baiting and iterative mapping approach
Source: Nucleic Acids Res. 2013 May 9;41(13):e129. doi: 10.1093/nar/gkt371 (PMC3711436; doi:10.1093/nar/gkt371)
Supplement: Supplementary Data [file supp_gkt371_nar-02763-met-k-2012-File007.pdf]

# Reconstructing mitochondrial genomes directly from genomic next generation sequencing reads - a baiting and iterative mapping approach

Christoph Hahn[1,*], Lutz Bachmann[1] and Bastien Chevreux[2]

[1] Natural History Museum, University of Oslo, Oslo N-0318, Norway
[2] DSM Nutritional Products Ltd., Department for Human Nutrition and Health, Kaiseraugst CH-4303, Switzerland

* To whom correspondence should be addressed. Tel: +47 22 85 18 27; Fax: +47 22 85 18 35; Email: christoph.hahn@nhm.uio.no

## Supplementary methods

### Proofreading algorithm introduction

We have developed a proofreading procedure to deal with mixed datasets which include mitochondrial genomes from more than one species, as in metagenomic datasets, if sample contamination is unavoidable or the data has been generated from a pooled sample of several species. The procedure is implemented in MITObim (option --proofread; at the moment only functional in instances with a single mitochondrial barcode to be used as seed reference) and requires a paired end sequence library.

### The origin of orphan reads in mapping assemblies

The term orphan read refers to the presence of only one member of a read pair in a dataset. Assuming both reads of a read pair have successfully passed initial quality control checks, orphan reads in MITObim mapping assemblies may occur due to three possible scenarios:

(a) the presence of conserved regions longer than the NGS read length but shorter than the library insert size, which are shared by two or more species represented in the dataset (i.e. mixed or contaminated DNA extracts) may cause the incorporation of heterologous orphan reads during the process of assembling the mitochondrial genome of a given species. Conserved regions exceeding the length of the library insert size will render the proofreading procedure useless.

(b) in the process of extending a given reference, one member of a read pair may map at the very edge of the reference, while the second member may lie in a yet unassembled region outside the current *contig*.

(c) an incorrect extension with a heterologous orphan will cause the rejection of correct reads at the *contig* edge. This may lead to incorrectly orphaned reads at a distance from the *contig* edge that is defined by the library insert size.

Proofreading algorithm details:

In order to minimize the risk of incorporating false positive heterologous reads, i.e. reads corresponding to any species except the one defined by a specific starting seed, the mapping assembly performed in step three of MITObim (see algorithm overview in the main paper) is restricted to perfectly matching reads with zero mismatches to the reference. Those reads which map perfectly to the reference (i.e. the potential mitochondrial reads of the species in question) are then assigned to two bins: one containing good pairs (both members of the read pair map) and the other containing orphans (only one member of the read pair maps perfectly). Orphan reads are likely to indicate an incorporation of heterologous reads. In a next step orphan reads are thus discarded based on their relative mapping position, i.e. if occurring at greater distance from the *contig* end than 2x the library insert size (orphan scenario a) or at the edge of the *contig*, within a distance from the *contig* end as defined by the read length (orphan scenario b). Orphans occurring between these limits may correspond to either orphan case (a) or (c). To discriminate between these cases the proofreading procedure subsequently searches for regions with substantially increased coverage (as observed for conserved region). The read coverage is assessed for every base within a 2x library insert size distance from the *contig* end. To detect unusual coverage fluctuations, e.g. a sudden increase / decrease in coverage at the beginning / end of a conserved region, the standard deviation of per base coverage across 10 bp sliding windows is calculated. Windows with significantly changing coverage, causing the standard deviation to exceed an empirically determined value of 6, are identified. Regions between the two outermost such windows are screened further for coverage peaks exceeding an empirically determined value of 1.6x average coverage. If detected, such regions are flagged as putatively conserved and orphan reads occurring in such regions are discarded (scenario a orphans). Any orphans remaining after this procedure (putative scenario c orphans) are resurrected, i.e. reintegrated as full pairs into the good pairs bin. In a final step, the mapping assembly is repeated using exclusively this paired read pool.

Note: A prerequisite for a successful discrimination of coverage fluctuations caused by conserved regions from stochastic coverage fluctuations is an even coverage across the contig as well as a roughly even representation of all species represented in the dataset. The success of the procedure for discriminating different mitochondrial genomes is furthermore highly dependent on the read length and insert size of the sequencing library. The procedure is likely to fail if applied to datasets which contain high amounts of PCR duplicates or reads from species sharing prolonged conserved sub-sequences of length exceeding the insert size of the library. For any species pair used in case study four, the longest conserved sub-sequence between the mitochondrial genomes of two species was found to be on average 235 ± 55 bp, i.e. exceeding the simulated read length (150 bp) in all cases. The length of the longest conserved sub-sequence is however a parameter which does not always follow a linear relationship with phylogenetic relatedness and is therefore hard to predict. In case study four, for instance, it reaches its maximum between *Salvelinus alpinus* and *Salmo trutta* (328 bp), and not between the congeners *S. salar* and *S. trutta* (267 bp).

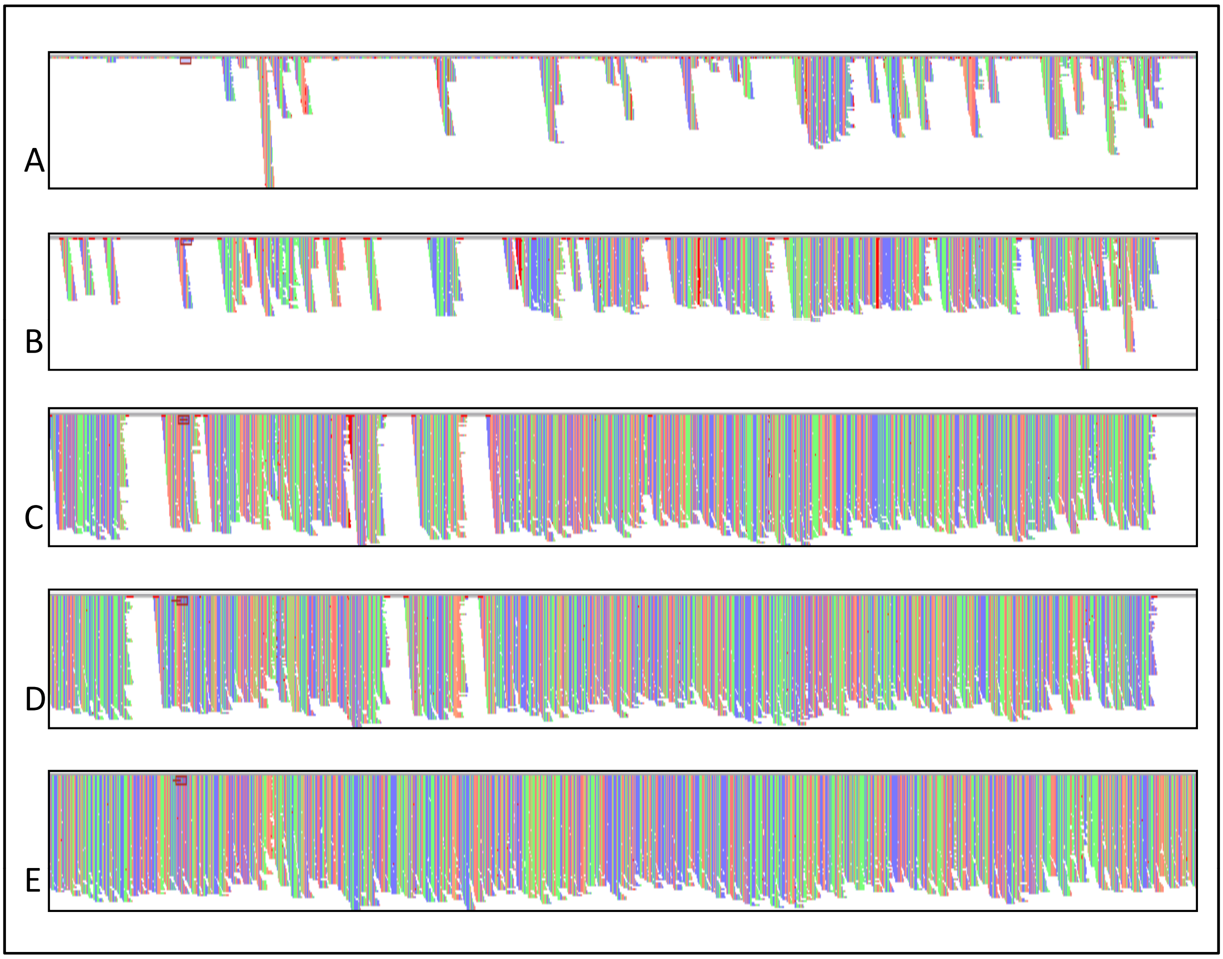


Supplementary figure 1. Example illustrating the MITObim process for *Gyrodactylus thymalli* (dataset "thy") using the mitochondrial genome of *G. derjavinoides* as a starting reference. A – result of the initial mapping assembly. B – result after 1st iteration. C – result after the 5th iteration. D – result after the 8th iteration. E – result after the 15th iteration. Snapshot taken from Tablet (36).
